# Chromatin Organization by Repetitive Elements (CORE): A Genomic Principle for the Higher-Order Structure of Chromosomes

**DOI:** 10.3390/genes2030502

**Published:** 2011-08-02

**Authors:** Shao-Jun Tang

**Affiliations:** Department of Neuroscience and Cell Biology, University of Texas Medical Branch, Galveston, TX 77555, USA; E-Mail: shtang@utmb.edu; Tel.: +1-409-772-1190

**Keywords:** repetitive DNA, repetitive element, repetitive sequence, DNA repeat, chromosome, chromatin

## Abstract

Eukaryotic genomes contain a large amount of DNA repeats (also known as repetitive DNA, repetitive elements, and repetitive sequences). Here, I propose a role of repetitive DNA in the formation of higher-order structures of chromosomes. The central idea of this theory is that chromatin regions with repetitive sequences pair with regions harboring homologous repeats and that such somatic repeat pairing (RP) assembles repetitive DNA chromatin into compact chromosomal domains that specify chromatin folding in a site-directed manner. According to this theory, DNA repeats are not randomly distributed in the genome. Instead, they form a core framework that coordinates the architecture of chromosomes. In contrast to the viewpoint that DNA repeats are genomic ‘junk’, this theory advocates that repetitive sequences are chromatin organizer modules that determine chromatin-chromatin contact points within chromosomes. This novel concept, if correct, would suggest that DNA repeats in the linear genome encode a blueprint for higher-order chromosomal organization.

## 1. Introduction

In humans and other higher organisms, repetitive DNA sequences make up more than 50% of the genome [1-3]. Based on their distribution modes in linear genomes, repetitive DNAs are classified as tandem or dispersed (interspersed) repeats. Tandem repeats are organized as ‘head-to-tail’ arrays, while dispersed repeats are distributed as individual copies in the genome [2-4].

DNA repeats have been prevailingly regarded as genomic ‘junk’ or ‘selfish parasites’ because most, if not all, apparently do not encode functional proteins for the host cells [5,6]. However, some more recent studies have identified roles of specific repetitive elements in various genic processes, including gene evolution, gene regulation and recombination. These roles have been systematically and nicely reviewed [7-10]. Despite these significant findings, a universal framework for understanding the roles of repetitive DNAs as a whole is still lacking.

Here, a non-genic perspective is taken to understand the biological function of repetitive DNAs. Particularly in this paper, this problem is tackled from the angle of the spatial organization of DNA repeats in the nuclear space. The rationale behind this approach is that the specific spatial organization may reveal novel insights into the function of repetitive DNA in the cell. Towards this end, the published work from various lines of investigation relevant to the spatial organization of DNA repeats was considered. The results from this analysis indicated that repeats in the same family, as most clearly shown by dispersed repeats, tend to pair in the cell, and that repeat pairing (RP) results in spatial clustering of repetitive DNAs in the nucleus and chromosomes. These findings suggest a potential role of DNA repeats in coordinating the higher-order structures of chromosomes.

Focusing on the potential function of DNA repeats in chromosomal organization, this paper presents a theory that highlights the following key points: (1) chromatin loci with DNA repeats in the same family tend to pair; (2) repeat-repeat pairing drives site-directed folding and cross-linking of chromatin in chromosomes; and (3) RP-mediated dynamic chromatin folding and cross-linking delineate chromosomal plasticity. The theory proposed here may provide a molecular mechanism for functional organization of the chromosomes. The literature cited in this paper focuses on the points mentioned above and represent only a portion of many excellent studies in the field of repetitive DNA.

## 2. Repeat Pairing (RP)

The key argument of this paper is that chromatin segments with DNA repeats in the same family pair and thus are clustered in specific spatial domains in the nucleus. Somatic pairing of homologous chromatin is a ubiquitous phenomenon in eukaryotic cells. Converging lines of evidence support this notion. Some of the evidence is concisely summarized here. (1) *Cytological evidence*. In salivary gland cells of *Drosophila* larvae, the formation of polytene chromosomes results from homologous pairing of chromatin [11-13]. In *Arabidopsis* cells, homologous interphase euchromatin is associated [14]; (2) *Genetic evidence*. Homologous pairing is essential for various genetic events, including homologous recombination and transvection [14-20]; (3) *Molecular evidence*. *In vitro*, double-stranded DNA (dsDNA) fragments with identical sequences selectively assemble themselves into bundles of multiple dsDNAs under physiological cation (Mg^2+^) concentrations, even in the presence of non-homologous dsDNA [21]. This observation suggests that homologous dsDNA molecules adopt complementary conformations that promote self-assembly. Indeed, space-filling models demonstrated that homologous dsDNA may form multi-stranded DNA structures [22]. However, because of DNA packaging on nucleosomes in chromatin, chromosomal DNA in cells may not behave the same as the naked DNA *in vitro*. For repetitive DNA in chromatin to directly mediate RP in the cell, nucleosomes may need to be removed by chromatin remodeling mechanisms. Although many details are unknown, it is clear that DNA sequence homology is the key determinant in somatic homologous pairing.

The undisputed phenomenon of somatic homologous pairing, as outlined above, strongly suggests the existence of molecular and cellular mechanisms that support the recognition and interaction of chromatin regions with homologous DNA sequences. Bearing with this notion, I propose that chromatin loci with repeats that are in the same family interact in the cell and term this type of chromatin interaction ‘repeat pairing’ (RP). Here, RP is used to describe the association of chromatin regions containing homologous DNA repeats. Although direct DNA contacts via classical Watson-Crick base pairing is not assumed, recognition of the homology of DNA repeats is a key mechanism involved in RP.

Multiple lines of evidence support the idea of RP of repetitive DNA in the cells. This is most clearly indicated by the spatial clustering of dispersed repeats. For example, fluorescent *in situ* hybridization (FISH) revealed that dispersed repeats in the same family often cluster into globular chromosomal domains in metaphase chromosomes [23,24]. Clustering of dispersed repeats into globular domains has also been observed in interphase nuclei [25]. Because dispersed repeats are, in general, distributed as single copies in linear genomes [2], the clustered rather than diffused spatial organization of the repeats strongly suggests their association with one another in the nucleus (RP). RPs may occur intra-chromosomally (especially in metaphase chromosomes) or inter-chromosomally (especially between interphase chromosomes). In addition, FISH also revealed that in human chromosomes Alu- and L1-dispersed repeats were ‘condensed’ into discrete mitotic chromosome domains [26]. By analyzing the distribution of these repeats in the YAC clones corresponding to the chromosome domains, Porta *et al.* excluded the possibility that this ‘condensed’ spatial organization of Alu and L1 repeats in the mitotic chromosomes simply reflects repeat clustering in the linear genomic regions [27]. Similar spatial clustering was also reported for other dispersed repeats [28]. Furthermore, molecular mapping of *in vivo* chromatin proximity provided direct evidence for spatial clustering of homologous repeats. For example, repetitive genes encoding tRNAs and olfactory receptors (ORs) are dispersed in linear genomes, but they stay in close proximity to each other within the nucleus [29-31]. The studies of Tessadori *et al.* revealed that in cultured *Arabidopsis* cells, RPs are controlled by cell states [32,33]. RP is also thought to be responsible for various repeat-mediated cellular processes, including repetitive DNA silencing [34] and chromatin aggregation in the macronuclei of the ciliate, *Oxytricha nova* [35,36]]. Furthermore, RPs likely occur among tandem repeats within the same array and help them fold into dense structures *in vivo* [2,37].

How is RP established? It is probable that mechanisms similar to homologous pairing during meiosis and recombination are involved [38,39]. In addition, DNA repeats may intrinsically facilitate RPs. For instance, repeats often harbor non-B-form DNA structures, such as Z-DNA [40,41], which has an inherent tendency towards self-association [42,43]. Furthermore, proteins or protein complexes that can associate with multiple homologous DNA repeats may mediate RPs, as suggested by tRNA gene clustering [30].

RPs may be critically regulated by cations. In addition to neutralizing negative charges and removing repulsive forces, cations (especially the divalent cations Ca^2+^ and Mg^2+^) may establish salt bridges between interacting repeat chromatin regions. Cations may also facilitate RP by stabilizing repeat Z-DNA [44]. Indeed, divalent cations are the key factors that determine the stability of DNA bundles formed *in vitro* [21] and repeat-formed heterochromatin [45]. Additionally, RPs may also be regulated by macromolecules, such as RNAs, ‘scaffold’ proteins (e.g., TOPO II and SMC), and repeat DNA-binding proteins [46-52], by their interactions with repeats.

RPs are likely constrained by steric and topological arrangements of the involved repeats in linear chromatin. Although the number of repeats in the same family can be big, a given member may only have a few pairing partners that are sterically and topologically suitable during any given chromosomal state. In theory, a decrease of physical distance between two repeats in linear genomes should promote their pairing. To facilitate RPs, repeats in the same family probably need to be located in the same vicinity within the genome. Consistent with this idea, several families of dispersed repetitive sequences are enriched in specific genomic segments [53-56]. Alu repeats that are inserted outside the Alu-enriched region are quickly eliminated [55].

Repeats in different families likely have distinct optimal conditions for pairing, due to their unique sequences, lengths, and linear genomic distributions. It is intriguing to envision that they have evolved to cluster in different cell states and thus generate physiologically relevant chromosomal conformations (see below).

## 3. RP in Chromosomal Organization

**Figure 1:**
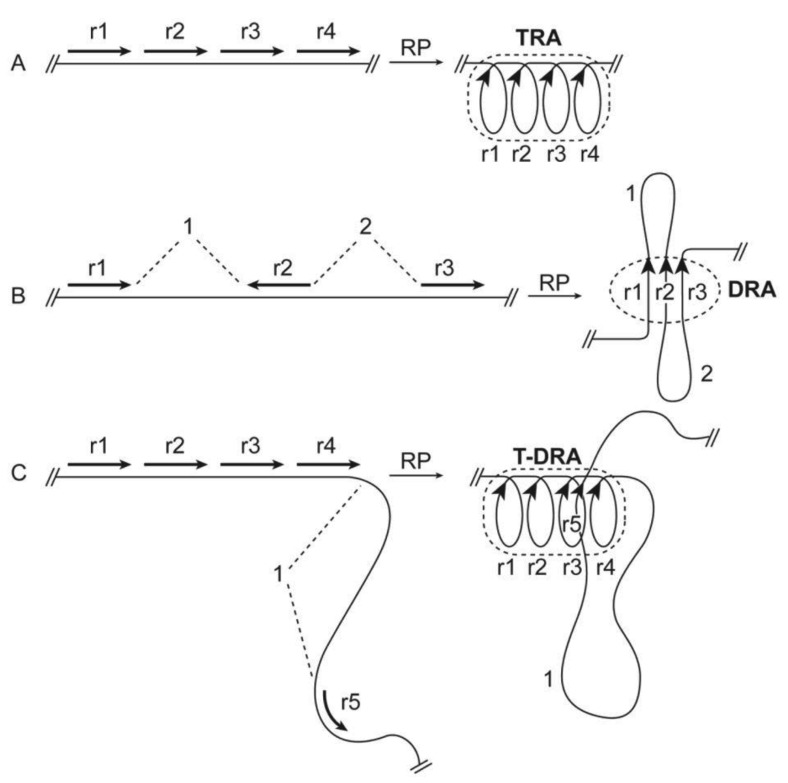
Schematic illustration of different modes of repeat pairing (RP) and RP-mediated chromatin folding. Lines represent chromatin fibers and arrows represent repeats and their orientation on chromatin. Dashed-line loops highlight the paired structures of repeat assemblies (RAs). Although fiber-fiber pairing is depicted in the diagram, it is meant to emphasize the association or interaction of the homologous repeats, rather than the details of pairing. (A) RP among repeats in a tandem array (r1-r4) causes the formation of a tandem repeat assembly (TRA) with a solenoid structure in which adjacent repeats are paired. (B) RP among dispersed repeats (r1-r3) leads to the formation of a dispersed repeat assembly (DRA) and loops from inter-repeat segments (1 and 2). (C) RP among tandem repeats (r1-r4) and their dispersed homologs (r5) results in the formation of tandem-dispersed repeat assembly (T-DRA) and loops (1).

The second critical idea of this paper concerns the consequence of RP and spatial repeat clustering on chromatin organization. RP-directed spatial clustering of DNA repeats would clearly cause re-distribution of involved chromatin regions. I propose that RP is a driving force that specifies the higher-order chromatin organization. As two repeats pair, they must fold or crosslink chromatin at the chromatin regions harboring the repeats (Figure 1). In other words, the formation of RPs drives chromatin association in a site-directed manner. Under this framework, DNA repeats function as matchmakers that specify the chromatin contact points, and their coordinated pairing governs chromatin folding and cross-linking. If this view is correct, the repeats in a genome would encode an internal logic or blueprint for the higher-order organization of chromatin. When the linear distribution of repeats in chromatin is determined, the potential higher-order organization of the chromatin is also largely determined. As such, repeat elements may act as chromatin organizer modules. These ideas are the basis for the theory of Chromatin Organization by Repetitive Elements (CORE). Next, I shall illustrate in more detail the key features of the CORE theory and relevant supporting evidence.

Repeats may organize chromatin into various conformations via different modes of RP-based folding (Figure 1). RPs within a tandem repeat array would intuitively fold chromatin into a solenoid conformation [37] (Figure 1A). In support of this idea, satellite DNA with multiple tandem repeat units developed solenoid tertiary structures *in vitro* [57,58]. On the other hand, RPs among *cis*-dispersed repeats would fold chromatin into loops (Figure 1B). Thus, this type of RPs can provide a sequence-directed mechanism for loop formation. Dispersed repeats may also cause loop formation by pairing with homologous tandem repeats (Figure 1C). Previous work identified scaffold/matrix attachment regions (SARs/MARs) as *cis* elements for radial loop formation [50]. Interestingly, SARs are AT-rich repetitive motifs with high homology to satellite repeats [50].

RP-generated chromatin loops are closed circular structures and thus may facilitate further chromatin condensation by supercoiling. This supercoiling process might be facilitated by other factors such as condensins, which bind to structured DNA and have helicase activity [59-61]]. Thus, RPs in theory can promote both chromatin folding and supercoiling, two fundamental processes in chromosome packaging [62]]. RPs among repeats in different loops conceivably create chromatin networks within chromosomes.

Intuitively, as a result of RP, repeats may form a compact structure (likely with associated proteins and RNAs), which is termed here as repeat assembly (RA). RAs constitute a dense core structure that organizes loose chromatin fibers (Figure 1). This idea will be developed further in a separate paper. Packaging of DNA repeats into dense heterochromatin has been demonstrated by various approaches [45,63-73]. This notion is also consistent with two other observations: (1) the spatial clustering of dispersed repeats in chromosomes [23-25]; and (2) tandem-repeat-based formation of densely packed chromomeres of chicken lampbrush chromosomes [74].

As indicated by polytene chromosomes and homologous DNA bundles *in vitro* [21,75], multiple (>2) repeats can be assembled into the same RA. The RA size is probably determined by the available homologous repeats and the intrinsic mechanistic constraints of the RPs. For multiple repeats to assemble an RA, at least some of the repeats must have more than one pairing partner. Several mechanisms can be envisioned. One possibility is that a repeat has more than one interaction surface that can accommodate multiple partners. A second possibility is that a repeat uses different sub-regions to pair with different partners. A third possibility is that macromolecules, such as proteins and RNAs, mediate the interaction of one of the repeats with different partners, as suggested previously [76] and exemplified by condensin-mediated tRNA gene clustering [30]]. The mode of RP in which one repeat simultaneously interacts with multiple other members would increase the chromatin packaging power of repeats.

The stability of RAs may vary according to the nature of the repeats involved. For example, some satellite tandem repeats may form very stable RAs, and thus would be frequently found in heterochromatic regions [64,65]. RA stability is presumably regulated by the ionic environment and the cell state [45,71-73]. Therefore, it is unlikely that all repeats are in RAs during any given cell state. Different sets of repeats may form RAs to generate distinct biologically-relevant chromosomal structures under different physiological conditions.

Some families of repetitive elements (e.g., Alu) are very abundant, and are distributed throughout the human genome. One may conceive that such elements are probably not suitable to for chromosome organization, because they might face higher stochastic noise in choosing paring partners. However, as proposed earlier, RP is likely constrained by other factors, including steric and topological limitations; these factors may restrict RPs for a specific element to a small number of specific pairing partners in a given chromosomal conformation. In addition, proteins or RNAs may also contribute to constrain RP.

## 4. Dynamic RP and Chromosomal Plasticity

In cells, chromosomal structures undergo plastic changes, and such structural changes are critical for coordinating genome activity [76]. However, the mechanism governing chromosomal plasticity is unclear. The CORE theory predicts that dynamic formation and disruption of RPs is a primary force that drives the structural plasticity of chromosomes.

Ample evidence indicates dynamic assembly and disassembly of repeats in the cell. For example, specific tandem and dispersed repeats in the *Arabidopsis* genome display distinct clustered or dispersed organization in the nucleus in response to different cell states [32,33]. In the yeast *Saccharomyces cerevisiae*, repetitive tRNA genes are distributed throughout the genome, but they are spatially clustered in the nucleus in a transcription-dependent manner [29,30]. Although repetitive olfactory receptor genes are scattered throughout mammalian genomes, they are spatially clustered during transcriptionally inactive states [31]. In mammalian cells, dispersed repeats form closed, compact chromatin structures that can be transiently opened by stress stimulation, such as heat-shock or viral infection. These closed and open states of the repeat chromatin can be rapidly inter-changed [71-73].

It is evident that assembling and disassembling of RAs via dynamic RPs would cause re-organization of the chromosomes. In support of a role for repeats in chromosomal plasticity, Alu, Ty2, and rDNA repeats are in the binding regions of specific chromatin remodeling complexes [77,78]. Although the involvement of other potential processes cannot be excluded, RP dynamics provide a simple and accurate molecular mechanism for reversible chromosomal plasticity.

The RP-driven chromosome plasticity may not occur in a stochastic manner. Conceptually, this plasticity may be programmed by sequential RPs. I speculate that, by altering steric and topological constraints, preceding RPs would critically influence subsequent RPs. As such, sequential RPs may restrict chromosome plasticity to specific pathways. This RP-based programming should not be viewed as rigid because a given repeat may have more than one potential pairing partner. Similar to RP regulation, multiple factors, including cations (Mg^2+^ and Ca^2+^), ‘scaffold’ proteins (TOPO II and condensins), epigenetic modifications, repeat-binding proteins, and transcriptional activity, may modulate chromosome plasticity.

If the idea of RP-guided plasticity is correct, it predicts that a chromosome with repeats can adopt different conformations. Each of the conformations is specified by a given set of RPs. Therefore, it follows that the more repeats chromatin harbors, the more conformations a chromosome can potentially adopt. This insight implies that repeat replication during evolution may enhance the structural plasticity of chromosomes.

## 5. Challenges and Implications

Several important questions remain unanswered. One is the mechanistic nature of RPs. Both direct and indirect RPs can be imagined. Direct RP probably requires structural complementarities of the repeat chromatin and are mediated by weak interactions, such as salt bridges and hydrogen bonds. In this scenario, direct contacts between interacting repeats likely occur. As indicated by *in vitro* studies of dsDNA aggregation [21], such direct repetitive DNA interactions may occur when nucleosomes are excluded from the chromatin regions. On the other hand, by simultaneously binding to two or more repeats in the same family, macromolecules, such as proteins and RNAs, may mediate RP indirectly. For instance, MeCP2 can simultaneously bind to spatially separate SARs and bring them together [46,47]. Direct and indirect interaction modes are not mutually exclusive and may be used by different repeats. At this stage, sufficient data are not available to convincingly argue that RP occurs on chromatin regions with and/or without nucleosomes.

Although it is easy to envision that the coordinated action of RPs in different repeat families determines the architecture of chromosomes, the specifics of RP-based chromosomal morphogenesis are unknown. Because the morphology of a given metaphase chromosome is reproducible in different cells and reversible *in vitro*, RPs likely are constrained to specific pathways in which one repeat can only pair with limited partners among many potential ones in the same family. Thus, specific biophysical rules, which are presently unclear, must govern the selection of specific partners. Understanding these rules could allow one to begin to predict the tertiary structure of chromosomes based on the distribution of DNA repeats. Recent technological advances for mapping chromatin proximity *in vivo* may have paved the way to elucidate these rules [79]. In addition, mining of the existing data sets from chromatin interaction mapping may provide clues about RP.

This paper argues for a critical role of RP in organizing eukaryotic chromosomes. The CORE theory predicts that disruption of RP would disturb higher-order chromosomal architecture. In this context, it is interesting to reconsider the previous observations made with the AT-hook protein, MATH-20 [80]. MATH-20 preferentially binds AT-rich sequences, which are characteristic of satellite tandem repeats and SAR repeats. Thus, it could conceivably coat AT-rich sequences and interfere with RPs among them. Strikingly, MATH-20 incubation can collapse mitotic chromosomes and transform them from their characteristic longitudinal and rod shapes to spherical structures [80]. This finding appears to support a role of AT-rich repeats in maintaining or establishing mitotic chromosomal structures. Eukaryotic genomes vary widely in repeat content. It is interesting to speculate, according to the CORE hypothesis, that chromosomes with low repeat content would adopt a relatively loose high-order organization. These low-repeat chromosomes would contain large chromatin segments that lacked repeat elements and would not assemble into tight RAs, thus resulting in higher mobility in the nucleus.

Although this paper focuses on the potential role of RP in chromatin organization, RP is probably a genomic event with broad functional implications and may provide a general conceptual framework for understanding the diverse biological activities proposed for specific repeats [7,8]. For example, RP may bring genes that are separated in the linear genome into the same spatial domain for co-regulation to support specific cell functions. One clear example of such co-regulation is illustrated by repetitive tRNA genes. Although there are 274 tRNA genes scattered throughout the linear yeast genome, they are clustered in the nucleus in a transcription-complex-dependent manner [29]. Interestingly, in support of the idea of repeat clustering-based co-regulation, the spatial clustering of tRNA genes contributes to the co-repression of their nearby genes [30]. In addition, a recent analysis of gene expression in mouse oocytes and preimplantation embryos revealed that different transposable elements are associated with synchronous expression of different sets of proximal genes at specific developmental stages [81]. For instance, mouse transcript (MT) retrotransposons are predominantly associated with genes expressed in oocytes, while MuERV-L retrotransposons are mainly associated with genes expressed in two-cell stage embryos. These observations suggest that repeats in the same family contribute to the temporal co-regulation of different genes and that the repeat-mediated synchronous expression of specific proximal genes is critical for mouse embryogenesis.

Although the proposed hypothesis is consistent with many published observations, it requires further direct supporting evidence. The central element of the hypothesis is RP. This idea may be proven or disproven by experimental determination of the incidence of homologous repeats in the spatial proximity to specific repeats in nuclear space. If the measured incidence is higher than a random expected distribution, it would support the author's hypothesis; otherwise, it would disprove the hypothesis. Recently developed technologies that probe internal chromatin contact points and chromosomal conformation in the nucleus have paved the way for such experimentation [31,79]. However, for reasons discussed earlier, one should not expect that all repeats are within the proximal nuclear domains of their homologs (assembled in RAs) in a given cell state. This may present an experimental challenge in choosing the specific repeat element for testing. In addition, other approaches such as FISH will be very helpful for visualizing the assembly and disassembly of RAs in different cells or under different experimental conditions.

Repetitive DNA is the most dynamic component of eukaryotic genomes during evolution. The complex of tandem repeats can undergo lengthening or shortening, and dispersed repeats may be generated at or deleted from specific genomic loci. The CORE theory predicts that evolutionary dynamics of the structure and distribution of DNA repeats would have a direct impact on the chromatin organization in chromosomes. One may conceive that such dynamic changes of DNA repeats may destabilize chromosome organization and thus argue against a critical role of RP in maintaining chromosome structure. However, according to the CORE theory, a structure of chromosome is determined by the total RP in the chromosome. The ‘quick’ changes of small fraction of repeats during evolution should not have a drastic effect on chromosome organization. In addition, some ‘old’ repeats that are ‘fixed’ in the genome may play more important roles in maintaining a relatively stable organization. Nevertheless, at this stage, it is hard to delineate the details of a mechanistic correlation between repeat dynamics and chromosome organization. It is also important to note that the dynamic changes of repeats may provide a mechanism to introduce necessary changes of chromosome organization during evolution.

## 6. Conclusions

In the CORE theory, a DNA repeat-based genomic code for the higher-order organization of eukaryotic chromosomes is put forward. According to the theory, repeats govern chromatin organization in chromosomes via RP. Because of RP, DNA repeats are assembled into core clusters (RAs) that coordinate chromatin folding. In this scenario, DNA repeats are the organizer modules that specify the internal architecture of chromosomes. The organizer activity of repeats does not depend on genic functions but arises from their pairing, which generates a driving force for the folding of chromatin in a site-specific manner. By guiding (and mediating) site-directed chromatin folding, DNA repeats collectively encode an internal logic (or structural memory) for packaging lengthy chromatin in an orderly and reproducible manner. With the implementation of repetitive elements in chromatin, evolution has created a straightforward molecular mechanism for packing complex genomes in the limited nuclear space. However, it is important to point out that although the CORE theory proposed here emphasizes a role of RP among repetitive elements as an intrinsic genomic mechanism for coordinating chromatin organization in chromosomes, it does not exclude other possible mechanisms. In fact, additional macromolecules, such as ‘scaffold’ proteins and RNAs, may also play important roles in chromosome organization.

Most previous studies that aimed to understand the role of DNA repeats focused on searching for their genic functions. Though specific repeats were found to play roles in gene evolution and regulation, such specific functions are hard to generalize for all repetitive sequences. The novel concept of repeats as chromatin organizer modules provides a new perspective to appreciate the ‘gold mine’ of DNA repeats. Under this framework, DNA repeats are structural motifs that organize the genome. These repeats not only provide an architectural blueprint for chromosomes, but also implement this blueprint. If this view proves correct, the necessity of a large number of repetitive sequences to specify higher-order structure and plasticity of chromatin is obvious, and the seemingly ‘selfish’ replication behavior of repeats is probably a crucial step for structural re-organization of chromosomes during evolution.
